# An Analysis of Risk Factors for Hearing Function in Adults Living with Human Immunodeficiency Virus in Gauteng, South Africa

**DOI:** 10.1007/s12070-023-04375-z

**Published:** 2023-11-28

**Authors:** Ben Sebothoma

**Affiliations:** Department of Speech pathology and Audiology, University of the Witwatersrand, Private Bag X3, Wits, Johannesburg, 2050 South Africa

**Keywords:** Adults, HIV, Risk factors, Middle ear pathologies

## Abstract

The aim of this study was to explore risk factors for hearing loss or affecting hearing function in adults living with HIV. A quantitative cross-sectional design was employed. A non-probability purposive sampling method was used to select and recruit 132 participants aged 18 years and above from an HIV clinic within the Academic Hospital in Gauteng Province, South Africa. Participants’ hearing were tested using, video otoscopy, tympanometry, pure tones, and speech audiometry. Of the 22.73% prevalence of hearing loss in the sample, the multiple logistic regression, controlling for other variables, indicated that age (AOR) = 1.049; 95%CI: 1.0005 to 1.0978) (p-value = 0.048) and extended use of antiretroviral therapy (AOR) = 1.0073; 95%CI: 0.9312 to 1.0896)) (p-value = 0.856) were strongly associated with the development of hearing loss. Although the odds of male participants to have hearing loss was 2.3572 (95%CI: 0.9394 to 5.915) compared to females, this association was marginal (p-value = 0.068). Current findings provide evidence for the risk factors for hearing loss in adults living with HIV. Given that an extended use of ART and a higher number of CD4 are strongly associated with hearing loss, these findings raise important implications for a focused monitoring for this population in order to identify early signs of hearing loss and implement timeous intervention to reduce the potential impact of hearing loss.

## Introduction

The Human Immunodeficiency Virus (HIV) continues to be a public health concern worldwide. Although reports suggest that trends of HIV show a declining pattern globally, new infections of HIV continue to be identified [1]. In 2018, there was an estimated 1.7 million new HIV reported globally, with low- and middle-income countries (LMICs) comprising most of the infections [1]. Current reports suggest that there is approximately 40 million people living with HIV globally, with LMICs continuing to be the epicenter for the pandemic. In countries such as South Africa, HIV contribute to burden of diseases [2] and forms part of the quadruple burden of diseases [3], over and above a dilapidated health system [4], high levels of unemployment rates and poverty [5].

While antiretroviral therapy (ART) has been shown to be effective in treating and managing HIV [6], auditory and vestibular pathologies seem to persist and remain a challenge for individuals living with HIV [7, 8]. Extensive research has been conducted on hearing function in adults living with HIV [9–15]. Findings of these studies have indicated that hearing loss is common among adults living with HIV, with a prevalence ranging from 2.5 to 58% [8]. However, there is a dearth of evidence on risk factors associated with hearing loss in adults living with HIV, with a need to investigate the possible influence of these risk factors, including comorbidities, on development of hearing loss in this population.

Few studies have made connections between hearing loss and the number of CD4 cells [10, 16, 17]. These studies indicated that participants with low CD4 T cells are at a greater risk for developing hearing loss, suggesting a possible opportunistic infection that may result in conductive hearing loss (CHL) or hearing loss with conductive element. Some studies [13, 18, 19] have indicated that hearing loss in adults living with HIV may be due to the possible ototoxic nature of ART regimen. Mata and colleagues [13] found that the risk ratio for developing hearing loss on participants who are treated with highly active antiretroviral therapy (HAART) is double when compared to those who were not on treatment.

Some demographic characteristics of participants such as age, gender, and race have also been shown to be potential risk factors for hearing loss in the general population [20]. However, there are conflicting results about the influence of these factors in adults living with HIV. For example, Fokouo and colleagues [21] found that age, gender, CD4 cells and duration of HAART did not influence hearing loss in young adults aged 15 to 49 years in Cameroon. However, Torre and colleagues [11] found race to be one of the potential risk factors for hearing loss in adults living with HIV, while Obasineke and colleagues [17] found CD4 cell count to be a risk factor for hearing loss in adult population. These conflicting results, which may be due to methodological difference, different sample size and other factors, and the general dearth of evidence about risk factors, have necessitated the need for this study.

Khoza-Shangase [22] has also called for intensified audiological research, particularly in areas such as South Africa where HIV remains an epicenter. The author argued that research into HIV and audiology has clinical implications and can demonstrate the potential role of audiologists in assessment and treatment. Therefore, this current study intends to extend knowledge and understanding of the risk factors that increase susceptibility to developing a hearing loss in adults living with HIV.

## Aim

The primary aim of the study was to analyze risk factors associated with hearing loss in adults living with HIV.

## Objectives

- To describe all risks factors in a sample of adults living with HIV.
- To describe hearing function in a group of adults living with HIV.
- To establish if there is any association between risk factors and hearing function in adults living with HIV.

## Methods

This study employed a quantitative cross-sectional design. This method was deemed appropriate because data was collected at one point in time [23]. A non-probability purposive sampling was used recruit and select patients who meet the inclusion criteria [24]. Participants were recruited from an HIV clinic in Johannesburg, South Africa. The inclusion and exclusion criteria were similar to those in a previously published study [25], which specified that patients were eligible to participate in the study if they were diagnosed with HIV, attending the HIV clinic within the tertiary hospital, and were 18 years of age and older. Only one exclusion criterion was adopted and that was exclusion of adults who presented with otorrhea on the day of testing. The study commenced after ethical approval was secured from the Human Research Ethics Committee (Medical) of the University (Protocol number: M190752), and permission was granted by the hospital.

## Procedure

Following an ethical clearance, permission from the hospital, and informed consent from participants, all participants underwent a basic audiological test battery which included the case history collection and medical record reviews using a self-administered questionnaire and a data collection form, a video otoscopic evaluation using Firefly Wireless DE550, acoustic immittance testing using Titan 3.3 (Interacoustics, Denmark) and pure tone audiometry testing through the use of the GSI 61 audiometer (Interacoustics, Denmark). Air conduction thresholds were obtained between 0.25 Hz and 8 kHz using Sennheiser HA 200 supra-aural headphones, with cut-off normal hearing at ≤ 25dBHL. Bone conduction thresholds were also obtained between 0.25 Hz and 8 kHz through the Radio Ear B-70 bone conductor. The air/bone gap criterion used to define conductive hearing loss component was ≥ 10dB [26]. During testing, infection control measures were in place as required for audiological testing.

## Statistical Analysis

Raw data was converted into an excel spreadsheet which was created for the purpose of this study. A STATA version 15.2 was then used to analyze all the data. Both the descriptive and inferential statistics were used to analyze the data. Categorical variables were summarized using frequencies and percentages while continuous variables were summarized using median and interquartile range since data were not normally distributed. The normality assumption was assessed using the Shapiro Wilk test as well as the histogram plot with a superimposed normal curve. The association between hearing loss and risk factors was assessed using logistic regression. The univariate regressions to estimate crude measures of association and adjusted regression to account for the effects of other variables were used. Results were reported as odds ratio together with the corresponding 95% CI and the associated p-value.

## Results

Table 1 below summarizes the demographic characteristics of the participants. Of the 132 adults who participated in the study, there were 65% females and 35% males, with median age of 49 years and an interquartile range of 41 to 57.5 years. The minimum age was 18 years while the maximum age was 72 years. Majority of the participants had acquired secondary education (77.27%).

**Table 1:** Baseline characteristics of the participants

Variable	Categories	Frequencies	Percentages
Gender	Female Male	86 46	65.15 34.85
Ethnicity	Black White Coloured	127 1 4	96.21 0.76 3.03
Level of Education	Primary Secondary Tertiary	20 102 10	15.15 77.27 7.58
Year of ART diagnosis	1990-1995 1996-2000 2001-2005 2006-2010 2011-2015 2016-2020	2 13 32 28 25 32	1.52 9.89 24.24 21.21 18.94 24.24
Year patient initiated on ART	1995-2000 2001-2005 2006-2010 2011-2015 2016-2020	4 30 30 28 39	3.05 22.9 22.9 21.37 29.77
CD4 categories	<200 201-350 351-500 >500 Missing	18 28 23 57 6	13.64 21.21 17.42 43.18 4.55

**Fig. 1:**
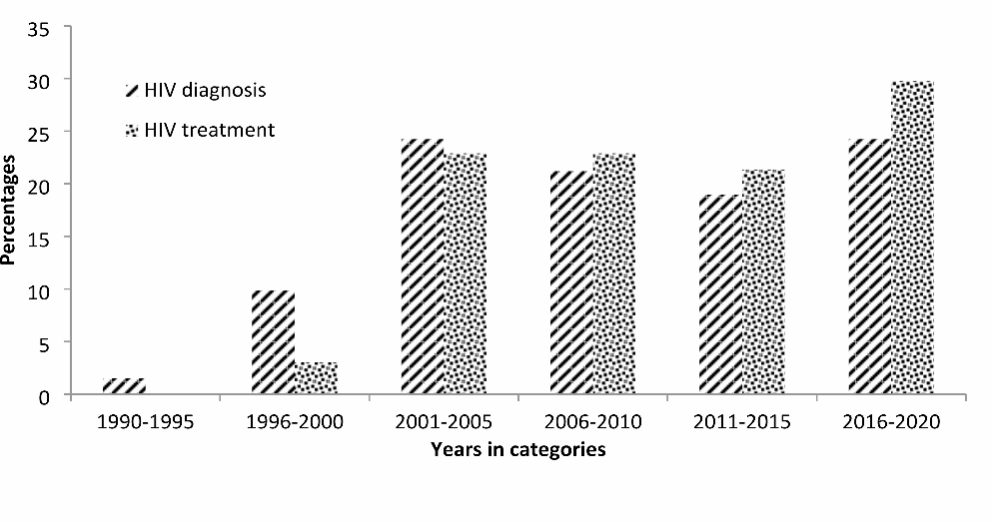
Distribution of ART diagnosis and ART initiation of the study participants

**Fig. 2:**
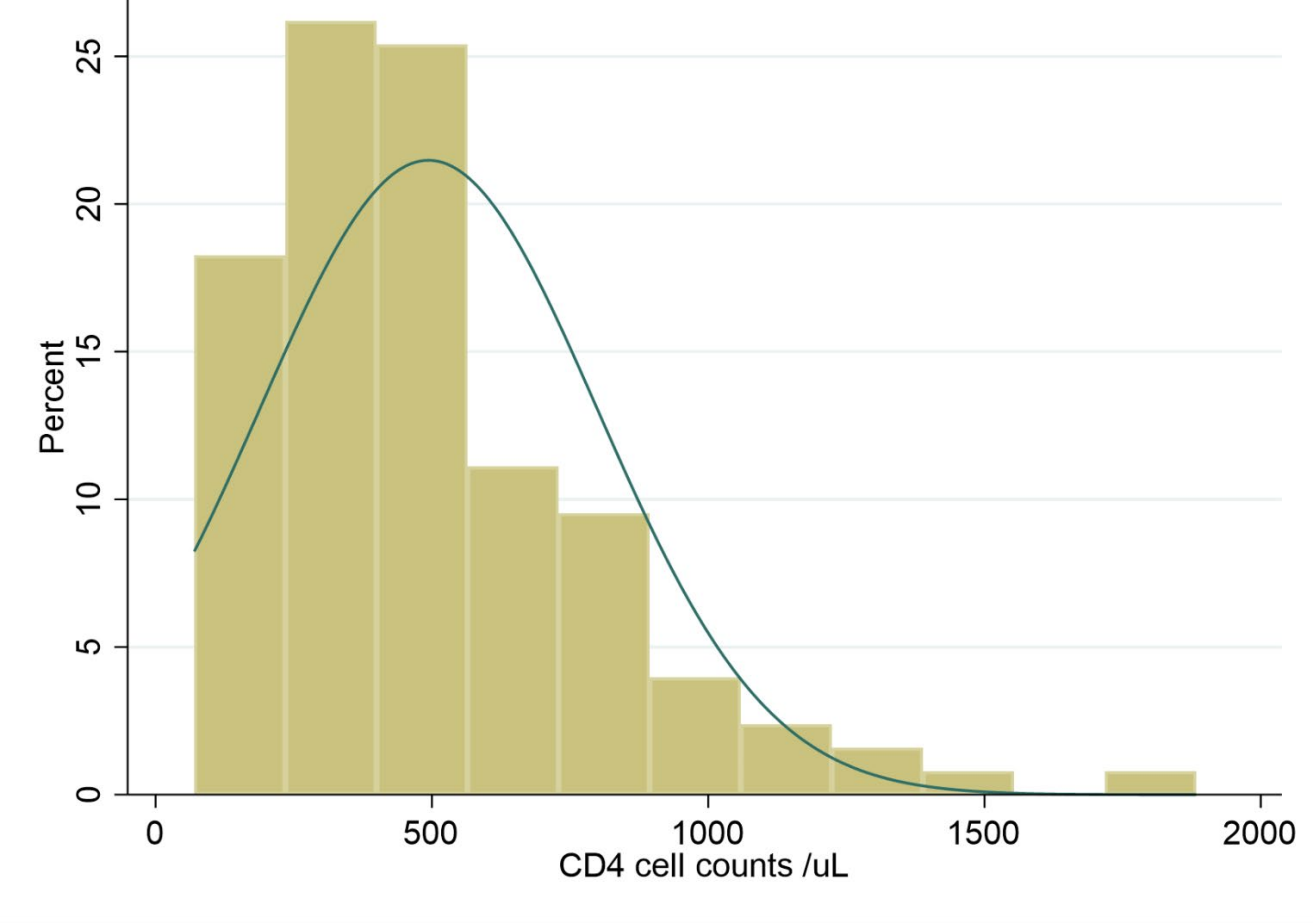
CD4 cell counts profile of the participants (n = 132)

Most of the participants were diagnosed with HIV between 2001 and 2005 (32%), while most were initiated on ART between 2016 and 2020. The proportion of the participants who were diagnosed with HIV was higher in 1990-2005 (47%) compared to the proportion of ART initiation in the same period (34%) (Fig. 1). The baseline CD4 cell counts were present in 95.45% of the participants. Most of the participants (43.18%) had a CD4 cell count above 500 cell/uL. The CD4 cell distribution and profiles are shown in Fig. 2 below. The median CD4 cell counts were 436 cell/uL with an interquartile range of 266-638 cell/uL.

There were 49.24% participants with comorbidities. Of those with comorbidities, 34.76% had a single underlying condition, 10.61% had two underlying conditions and 3.79% had three underlying conditions. The most common comorbidities were hypertension (23.48%), hypercholesterolaemia (21.21%), Anaemia (4.55%) and asthma (3.79%).

## Hearing Function in Adults Living with HIV

The prevalence of hearing loss in this cohort was 22.73%, with majority of the participants (77.28%) presenting with normal hearing function. Of those with hearing loss, 9.85% participants presented with unilateral hearing loss while 12.88% presented with bilateral hearing loss. The severity of hearing loss ranged from mild (21.21%), moderate (4.55%) to severe (9.09%). Regarding the left ear, 5.3% had a mixed hearing loss (MHL), 6.82% had a sensory/neural hearing loss (SNHL) and 7.58% had a CHL. Similarly, in the right ear, 3.79% had a MHL, 6.06% had SNHL and 6.06% had a CHL.

Participants who had hearing loss were older with a median age of 55 years compared to those with normal hearing who had a median age of 47 years (p-value = 0.0031). Participants with hearing loss had a higher median cell count of 517 cells/uL compared to those with normal hearing who had a median cell count of 423 cell/uL. However, there was no significant difference (p-value = 0.1403). Furthermore, those with hearing loss had a longer median ART duration of 11 years compared to participants with normal hearing with median ART duration of 9 years.

Among the participants with hearing loss, majority of them (53.33%) were females, with secondary level of education (70%) and 53.33% had comorbidities. However, none of these proportions were significantly difference from those HIV patients who did not have hearing loss (p-value > 0.05). About 33.33% of the participants with hearing loss were hypertensive, while 30% of those with hearing loss had hypercholestroremia. However, these proportions were not significantly different from those of the participants with normal hearing.

Table 2 shows results from bivariate analysis and logistic regression to determine the association between hearing function and risk factors. The univariate logistic regression to quantify factors associated with hearing loss among participants showed that a one-year increase in age among HIV patients increased the odds of having a hearing loss by 6.4% (OR = 1.064; 95%CI: 1.019–1.109) and this was statistically significant (p-value = 0.004). A one cell increase in CD4 cell counts among HIV patients increased the odds of having a hearing loss by 0.1% (OR = 1.001; 95%CI: 0.999–1.0003) and was marginally significant (p-value = 0.063). A one-year increase on ART among HIV patients increased the odds of having a hearing loss by 1.13% (OR = 1.0113; 95%CI: 0.999–1.0805) and was not significant (p-value = 0.74). Though the male participants had high odds of having a hearing loss, which was 1.914 (95%CI: 0.834 to 4.391) times higher compared to the female participants, this was not statistically significant (p-value = 0.125).

**Table 2:** Logistic regression of hearing outcomes and risk factors adjusting for the effects of other variables

Variable	Bivariate analysis			Univariate		Multiple regression	
	HF No n (%)	HR Yes n (%)	P-value	OR((95%CI)	P-value	AOR (95%CI)	P-value
Age Median (IQR)	47(39-56)	55(46-61)	0.0031	1.064 (1.019 to 1.109)	0.004	1.0479 (1.0005 to 1.0978)	0.048
CD4 cell count Median (IQR)	423(262-641)	519(352-638)	0.1403	1.001 (0.999 to 1.0003)	*0.063*	--------	-------
ART duration in years Median (IQR)	9(3 -15)	11(5 -15)	0.5549	1.0113(0.9465 to 1.0805)	0.740	1.0073(0.9312 to 1.0896)	0.856
Sex Female Male	70(68.63) 32(31.37)	16(53.33) 14(46.67)	0.122	reference 1.914 (0.834 to 4.391)	0.125	reference 2.3572(0.9394 to 5.915)	*0.068*
Education Primary Secondary Tertiary	12(11.76) 81(79.41) 9(8.82)	8(26.67) 21(70.0) 1(3.33)	0.101	reference 0.3889(0.141 to 1.073) 0.1667(0.018 to 1.583)	*0.068* 0.119	reference 0.4227(0.1409 to 1.2679) 0.1932 (0.0186 to 2.0022)	0.124 0.168
Comorbidities No Yes	53(51.96) 49(48.04)	14(46.67) 16(53.33)	0.610	reference 1.236(0.5467 to 2.795)	0.610	reference 0.7759(0.2421 to 2.4869)	0.669
Number of comorbidities 0 1 2 or more	53(51.96) 36(35.29) 13(12.75)	14(46.67) 10(33.33) 6(20.0)	0.606	reference 1.0516(0.4211 to 2.6263) 1.7473(0.5629 to 5.4226)	0.914 0.334	-------- --------	-------- --------
Hypertension No Yes	81(79.41) 21(20.59)	20(66.67) 10(33.33)	0.148	reference 1.928(0.7856 to 4.7345)	0.152	reference 1.4315 (0.4031 to 5.0841)	0.579
Hyper-cholesterolaemia No Yes	83(81.37) 19(18.63)	21(70.0) 9(30.0)	0.180	reference 1.8722(0.7413 to 4.7281)	0.185	--------	--------

*HF* = hearing function

Participants who went to secondary school were 62% less likely to have a hearing loss compared to those who have primary level education (OR = 0.3889; 95%CI: 0.141 to 1.073) and this was marginally significant (p-value = 0.068), while those who had tertiary education were 83% less likely to have a hearing loss (OR = 0.1667; 95%CI: 0.018 to 1.583) and this was not statistically significant.

The univariate analysis showed a crude odds of 1.236 (95%CI: 0.5467 to 2.795) for the comorbidity covariate. This means that those participants with comorbidities were 1.23 times more likely to have hearing loss compared to those who did not have comorbidities; However, this was not statistically significant (p-value = 0.61). Though there was a dose-response effect on the number of comorbidities with the developments of hearing loss, the association was not statistically significant (p-value > 0.05). The more the number of comorbidities the patients had the higher the odds of developing hearing loss compared to those without any comorbidity. Those who had three underlying conditions were 2.52 times more likely to develop hearing loss than those without any condition. Hypertensive patients were 93% time more likely to develop a hearing loss compared to those who were not hypertensive (OR = 1.928; 95%CI: 0.7856 to 4.7345). However, this was not statistically significant (p-value = 0.152). Similarly, participants with hyper-cholesterolaemia were 87% times more likely to develop hearing loss compared to those without hyper-cholesterolaemia, but this was not statistically significant (p-value = 0.185).

In the multiple logistic regression model, a significant association was observed for the age variable. A one-year increase in age increased the odds of having a hearing loss by 4.79% (adjusted odd ratio (AOR) = 1.049; 95%CI: 1.0005 to 1.0978) (p-value = 0.048) adjusting for the other variables. A one-year increase in ART duration increased the odds of having a hearing loss by 0.73% (adjusted odd ratio (AOR) = 1.0073; 95%CI: 0.9312 to 1.0896)) (p-value = 0.856) adjusting for the other variables. Male participants had an increased odds of having a hearing loss of 2.3572 (95%CI: 0.9394 to 5.915) compared to females which was marginally significant (p-value = 0.068) while adjusting for other factors. No significant findings were observed for level of education (AOR = 0.4227; 95%CI: 0.1409 to 1.2679 for secondary level of education and AOR = 0.1932; 95%CI: 0.0186 to 2.0022 for tertiary level of education). Being hypertensive increased the odds of having a hearing loss by 1.43 times (95%CI: 0.4031–5.0841) compared to participants without hypertension. However, this was not statistically significant (p-value = 0.579). Participants who had other comorbidities were 33.4% (AOR = 0.7759; 95%CI: 0.2421 to 2.4869) less likely to have hearing loss compared to those who did not have hearing loss, and this was not statistically significant (p-value = 0.669).

## Discussion

The purpose of this study was to analyze risk factors affecting hearing function in adults living with HIV. This is one of the few studies that explored specific risk factors for hearing loss in adults living with HIV. Findings of the current study indicated that there are a number of risk factors that are individually associated with hearing loss in adults living with HIV. While some risk factors such as gender were also associated with hearing loss, the significance was marginal. However, age, extended use of ART, increased number of CD4 cell and some comorbidities were found to be significantly associated with hearing loss in this cohort.

Age was one of the common risk factors that was found to have a significant association with hearing loss in adults living with HIV. The findings indicate that adding one year increases the odds of developing hearing loss in adults living with HIV. These findings are consistent with findings of the study conducted by Torre and colleagues [11] who also found that age in adults living with HIV was significantly associated with higher pure tone average (PTA). This ageing as a risk factor needs to be interpreted with caution, particularly because hearing loss is often associated with ageing [27]. In fact, Sousa and colleagues [28] reported that presbyacusis begins around the fifth decade of life. Given that participants with hearing loss in this study had a median age of 55 years, it may be these participants were already having early onsets of age-related hearing loss. It is not surprising that Fokou and colleagues [21] did not find any significant association between age and hearing loss with their participants’ age ranged from 15 to 49 with mean age of 33.4 years. The lack of a control group in this study has made it difficult to compare and determine if there is any difference between groups. Therefore, future studies need to include the control group and perhaps employ designs such as case-control to establish the difference between groups.

Despite the lack of a control group in this study, current findings raise important implication for audiologists and other hearing health professionals to be cognizant of, and provide a tailor-made audiological assessment and management to adults living with HIV who are above the age of 50 years. Sousa and colleagues [28] reported that certain medical conditions such as diabetes may exacerbate or predispose patients to early onset of age-related hearing loss. It is not yet known that HIV predisposes patients to early onset and development of hearing loss, which require further exploration, but professionals must be cognizant of patients living with HIV.

Current findings indicates that one year increase on using ART increased the odds of having hearing loss. This means that the more patients use ART to manage HIV, prevent the mortality associated with HIV, and improve quality of life, the higher the odds of developing hearing loss. These findings seem to point to the potential ototoxic nature of the ART. These findings are consistent with previous studies that indicated that ART may potentially be ototoxic [14, 18]. Therefore, implication for clinical practice, policy and resource distribution are raised in these findings, suggesting the need for a continued and a focused ototoxicity monitoring program for adults living with HIV [29]. Future studies are needed to focus on the frequency of testing patients who are on ART in order to determine the contextually appropriate protocol.

The effects of gender on hearing pathologies have been studied in the literature, with male gender having the greatest risk of developing hearing loss. Although results were marginally significant, current findings indicate that male participants were more likely to have hearing loss than females. These findings seem to be consistent with published research, which indicated that male participants are at a greater risk for developing auditory pathologies such as middle ear pathologies. Therefore, these findings call for research to further explore the relationship between gender and hearing loss, and provide an understanding on the pathophysiology, particularly in adults living with HIV.

Another study which forms part of the bigger study conducted by the current author [25] using the same data has detailed the relationship between comorbidities and hearing loss in adults living with HIV. It is worth noting that in the current study the author briefly mentions these comorbidities as risk factors. Findings indicated that hypertension and hypercholestroremia were significantly associated with hearing loss. Findings also indicated that the higher the number of comorbidities, the higher the likelihood of developing hearing loss. Given that some of the comorbidities that contribute to the development of hearing loss fall under the quadruple burden of diseases, minimizing these comorbidities in adults living with HIV will reduce hearing loss and improve quality of life.

An increased number of CD4 T cells had increased odds of developing hearing loss the current cohort. Findings of the current study indicates that one cell increase in CD4 cell counts among HIV patients increased the odds of having a hearing loss. These findings are contrary to the findings of the studies such as Fokuou et al. [21] who found that CD4 cells did not have any influence on hearing function, but also on findings by Fasunla et al. [16], Obasineke et al., [17] and Van der Westhuizen et al. [10] who found participants with low CD4 cell count to be at a greater risk for hearing loss. The latter studies attributed hearing loss to opportunistic infection due to weaken immune system resulting from low CD4 cell count.

Given that participants with higher CD4 cell count were the ones with an increased risk for hearing loss, this could be due to the possible ototoxic nature of the ART alluded by Khoza-Shangase [14]. It is also not surprising that participants with an increased number of years on ART had an increased risk for developing hearing loss. Current author argues that the two risk factors, viz., duration on ART and higher CD4 cell count, explains the development of hearing loss in this population. Matas et al. [14] also found that participants who were exposed to ART had higher incidence of hearing loss. Findings of the study attributed the higher incidence of hearing to the potential ototoxic nature of the ART. Clinical implications for ototoxicity monitoring for patients who are living with HIV are raised. Longitudinal studies using comprehensive and sensitive measures for hearing function such as distortion product otoacoustic emission (DPOAE) and ultra-frequency audiometry to explore the change and the frequency of change for hearing must be employed in order to determine the ototoxicity protocol for patients living with HIV.

## Study Limitations

This study has provided some important information about risk factors associated with hearing loss in adults living with HIV. However, there are methodological limitations that need to be taken cognizance of during interpretation of the findings. First, the sub-sample of participants with hearing loss was small, limiting generalizability of the findings. For example, whilst some of the findings for the current study point to ototoxicity nature of HIV treatment, hearing loss with conductive element, which is also common despite the use of ART [34–36] require a further exploration. The small incidence of hearing loss in this sample makes it difficult to determine risk factors for hearing loss with a conductive element. Large scale studies are therefore needed to determine risk associated with hearing loss with a conductive element. Lastly, due to the fact that there is no control group in this study to make a comparison, there was no control for the influence of variables. These limitations raise implications for future studies.

## Conclusion

Current findings have indicated that hearing loss in adults living with HIV is common, and ageing, male, other comorbidities, increased number of CD4 cell counts, and extended use of ART increase the risk for hearing loss. These findings raise critical implications of preventive health care and preventive audiology to ensure that a careful attention is paid to adults living with HIV. Future research such as those that employ case-control or/and longitudinal design are needed to assist in determining the protocol that is tailored for adults living with HIV in order to identify any change in hearing that alter quality of life.
